# Effects of Intensive and Semi-Intensive Production on Sheep Milk Chemical Composition, Physicochemical Characteristics, Fatty Acid Profile, and Nutritional Indices

**DOI:** 10.3390/ani11092578

**Published:** 2021-09-02

**Authors:** Eleni Kasapidou, Zoitsa Basdagianni, Vasileios Papadopoulos, Chrysoula Karaiskou, Anastasios Kesidis, Arsenios Tsiotsias

**Affiliations:** 1 Department of Agriculture, School of Agricultural Sciences, University of Western Macedonia, 53100 Florina, Greece; vpapadopoulos@uowm.gr (V.P.); akesidis@uowm.gr (A.K.); atsiotsias@uowm.gr (A.T.); 2 Department of Animal Production, School of Agriculture, Aristotle University of Thessaloniki, 54124 Thessaloniki, Greece; basdagianni@agro.auth.gr (Z.B.); cnkaraisk@agro.auth.gr (C.K.)

**Keywords:** sheep milk, intensive production, semi-intensive, chemical composition, physicochemical characteristics, fatty acid composition, nutritional value

## Abstract

**Simple Summary:**

Intensive and semi-intensive sheep farming are the two farming systems usually employed in Greece. Several studies have been conducted on the effect of the farming system on sheep milk fatty acid composition. This study simultaneously examines milk basic composition, physicochemical characteristics, fatty acid composition, and nutritional value according to the production system. Bulk tank milk from farms using the intensive and semi-intensive production systems was analyzed. The results show that grazing can improve the fatty acid composition of milk. Thus, pasture-based feeding strategies could improve the fatty acid composition and the nutritional properties of sheep dairy products.

**Abstract:**

Dairy sheep farming is a vital sector of the agricultural economy in Greece. Information on the effect of the farming system on sheep milk characteristics is critical for producing dairy products with improved nutritional value according to the current guidelines for healthy food consumption. This study aimed to investigate the basic composition, physicochemical characteristics, fatty acid composition, and nutritional indices of milk produced in intensive and semi-intensive sheep farms. Bulk tank milk samples from 20 intensive and 20 semi-intensive sheep farms were examined. Sheep in the intensive farms were kept indoors and were fed with roughage, silage, and concentrates. Sheep in the semi-intensive farms were kept indoors during the night and were moved to the pastures during the day. Sheep were fed with roughage, silage, and concentrates in combination with grazing. Milk composition and somatic cell count were determined with automatic analyzers. The physicochemical characteristics were determined with standard laboratory methods. The fatty acid composition was analyzed by gas chromatography. The farming system did not affect milk chemical composition and physicochemical characteristics. However, milk fatty acid composition and nutritional value were significantly improved in milk from farms using the semi-intensive production system, and this favorable effect was attributed to the inclusion of pasture in sheep diet.

## 1. Introduction

Sheep breeding has a significant economic impact in the rural mountainous areas of Greece, and thus, there are 6.5 million dairy ewes [1] that produce approximately 650 million (tons) of milk annually [2]. The majority of sheep milk (90%) is converted into highly valued cheeses such as the well-known Feta cheese that is the most important export product of the Greek primary sector [3]. Sheep milk quality is primarily related to its processing performance, i.e., its capability to produce high yields of improved quality dairy products [4]. In this respect, information on the composition, physicochemical properties, and nutritional value of sheep milk is critical for the dairy industry and the quality characteristics of the dairy products. On the other hand, there is growing consumer interest in dairy products’ nutritional and health aspects [5]. Furthermore, the quality characteristics of sheep milk are also crucial for the farmers, since Greek dairy factories qualitative classify the produced milk and pay the farmers accordingly. The quality of sheep milk is affected by several factors such as genetics (breed and genotype), animal health, physiological (age and parity, lactation stage), environmental (season), and management (type of feed, farming system, milking techniques) aspects as well as their interactions [6,7,8].

With regard to the farming system, Hatziminaoglou et al. [9] classified the sheep production system in Greece as follows; (a) sedentary extensive, (b) transhumant, (c) semi-intensive, and (d) small intensive. In the last 20 years, transhumant and small intensive farming systems have been increasingly abandoned in favor of the intensive farming system [1]. However, the implementation of the intensive system results in higher feeding and structural expenses per ewe. Thus, a good alternative is a mixed feeding regime that combines grazing and a concentrate supplement [7,10]. Pasture feeding has a desirable effect on the flavor, antioxidant properties, and fatty acid profile of the produced dairy products [11,12,13,14]. According to Hadjigeorgiou [15], in Greece, more than 75% of the sheep farms are located in mountainous and less-favored areas where animal nutrition is still based on the use of the available rough grazing lands. Flocks graze natural pastures with native flora. According to the same author, the distribution of farms according to the farming system is 5% nomadic, 50% semi-extensive, 30% semi-intensive, and 15% home-fed. In the recent countrywide study of Lianou et al. [16] on milk hygiene from sheep flocks, the distribution of farms according to the production system was as follows: extensive 7%, semi-extensive 46%, semi-intensive 33%, and intensive 13%. As seen, the application of extensive and semi-extensive systems is declining, whereas the application of the semi-intensive system is increasing, and the application of the intensive system remains constant. Modern farmers prefer to keep their animals in facilities near their houses and feed them with either home-produced or commercial feeds while animals are allowed to graze in the nearby field. Finally, the semi-intensive farming system is popular in Greece because it allows farmers to lower the feeding costs.

The effect of the farming system on milk chemical composition, physicochemical characteristics, and fatty acid composition of commercially produced milk has not been extensively studied. Additionally, to our knowledge, no published data have simultaneously studied these groups of variables, enabling the production of milk with improved processing performance and a healthier lipid profile according to the current nutritional recommendations.

The objective of this study was to offer updated information on the chemical composition, physicochemical characteristics, fatty acid composition, and nutritional value of sheep milk produced in commercial farms using the intensive and semi-intensive farming system.

## 2. Materials and Methods

### 2.1. Sampling and Sample Processing

The study was carried out in dairy sheep farms located in the Regional Unit of Florina, Greece (40°46′58″ N 21°24′32″ E and altitude approximately 600 m) in the period from late May to late June 2019 and lasted 28 days. The Agricultural Veterinary Bureau of Aminteo supervised the farms. Farms were classified according to the European Food Safety Authority system [17]. Thus, animals in the intensive farms were kept in permanent housing with no access to pasture and were fed with roughage, silage, and concentrates. Animals in the semi-intensive farms were kept indoors during the night and some part of the day and were moved to the pastures during some period of the day. Sheep were fed with roughage, silage, and concentrates, in combination with grazing. The concentrates offered to the animals were either commercially produced, home-produced, or a combination of commercially and home-produced. Detailed information on the type of concentrate feed, grazing species and duration of grazing was not recorded. Sheep in all farms, participating in the study, were crossbred animals.

Bulk tank milk samples from 20 intensive and 20 semi-intensive sheep farms were collected. Dairy sheep farms were selected on a convenience basis, i.e., willingness of the farmers to collaborate in the study and to allow sample collection. Milk in the tanks was manually mixed with a plunger to achieve homogeneity before sampling. Samples (2 from each farm) were collected in 50 mL plastic screw-capped flasks, placed in isothermal containers with ice packs, and transported to the laboratory within 4 h following collection. Both samples were collected in the same day. Samples aimed for analyzing Somatic Cell Count (SCC), chemical composition, and physicochemical characteristics were kept at 4 °C and analyzed within 24 h following collection. Sodium azide (0.01 g/100 mL) was added as a preservative to the milk samples destined to determine Somatic Cell Count. Samples aimed for the determination of fatty acid composition were kept at −20 °C until analyzed.

### 2.2. Milk Chemical Composition and Somatic Cell Count

Milk composition (fat, protein, lactose, and total solids content) was determined by infrared analysis (FTIR interferometer), using a Milkoscan FT6000 Analyzer (Foss Electric, Hillerød, Denmark). Milk moisture content was calculated by deducting the total solids content from 100, and milk ash content was calculated by deducting fat, protein, and lactose contents from the total solids content. The ratio fat content/protein content was also determined. Somatic Cell Count (SCC) was determined with a Fossomatic 5000 instrument (Foss Electric, Hillerød, Denmark), and the obtained data were log-transformed to normalize the distribution.

### 2.3. Milk Physicochemical Properties

Before analysis, the samples were placed in a heated water bath to reach room temperature and, following that, they were thoroughly mixed by gentle inversion of the sample container multiple times without causing frothing. Milk pH was measured using a glass electrode with a built-in temperature sensor (5014T electrode, Crison, Barcelona, Spain) in a Crison GLP 21 pH-meter (Barcelona, Spain), which was calibrated with standard buffer solutions at pH 4.0 and 7.0 according to the manufacturer’s instructions. The electrical conductivity of the samples was measured by a GLP 31 conductometer (Crison Instruments, Barcelona, Spain) using a Sodium Ion-Selective Electrode 9650, calibrated with 147 μS/cm, 1413 μS/cm, and 12.88 mS/cm buffer solutions. Refractive index and Brix value were determined using a digital refractometer (DR6000-T, Krüss, Hamburg, Germany) set at 20 °C.

### 2.4. Milk Fatty Acid Composition

Fatty acid composition was determined by gas chromatography (GC) as described by Papaloukas et al. [18]. Briefly, lipids were extracted with a chloroform/methanol solution (1:2 *v/v*) that contained 0.01% (*w/v*) of t-butyl-hydroxytoluene (BHT) to prevent fatty acid oxidation during extraction, according to the Bligh and Dyer method [19]. Fatty acid methyl esters were prepared from the extracted lipids by base-catalyzed methanolysis of the glycerides using KOH in methanol, according to the method ISO-IDF 15884 [20] of the International Organization for Standardization. Fatty acid methyl ester analysis was performed on an Agilent Technologies 6890N GC equipped with a flame ionization detector (FID) and a 60 m × 0.25 mm i.d., 0.25 μm film thickness DB-23 (50% Cyanopropyl 50% dimethyl polysiloxane) capillary column (Model Number: Agilent 122 2362). The injector temperature was set at 250 °C. The oven temperature was programmed to increase from 110 °C (held for 6 min) to 165 °C at 1 °C/min (held for 13 min), to 195 °C at 15 °C/min (held for 22 min), and to 230 °C at 7 °C/min (held for 7 min). The carrier gas was helium at 0.7 mL/min, the injection volume was set at 3 μL, and the split ratio was 1:50. The injection was performed using an Agilent 7683 Series auto-sampler. Fatty acids were identified using three commercial standard mixtures: (a) 37-component FAME mix (Supelco, 47885-U), (b) PUFA-2, Animal source (Supelco, 47015-U), and (c) a mixture of cis- and trans-9,11- and -10,12-octadecadienoic acid methyl esters (Sigma-Aldrich, O5632-250MG) as reference standards. Fatty acids were quantified by peak area measurement, and the results were expressed as percent (%) of the total peak areas for all quantified acids. Fatty acids were grouped as saturated fatty acids (SFA), monounsaturated fatty acids (MUFA), polyunsaturated fatty acids (PUFA), and unsaturated fatty acids (UFA).

### 2.5. Milk Lipid Quality Nutritional Indices

The milk fatty acid profile was used to calculate the following indices related to healthy fat consumption. In addition, all nutritional indices were used to assess the nutritional value of milk and other dairy products in various studies.

The hypocholesterolemic/hypercholesterolemic fatty acid ratio (h/H) ratio was calculated according to the formula reported by Chen and Liu [21]:

h/H = (C18:1 *n*-9 *cis* + ∑PUFA)/(C12:0 + C14:0 + C16:0)

The atherogenicity (AI) and thrombogenicity (TI) indices were calculated according to the following formulae proposed by Ulbricht and Southgate [22]:

AI = [C12:0 + (4 × C14:0) + C16:0]/∑UFA

TI = (C14:0 + C16:0 + C18:0)/[(0.5 × ∑MUFA) + (0.5 × ∑*n*-6 PUFA) + (3 × ∑*n*-3 PUFA) + (*n*-3/*n*-6)]

The health-promoting index (HPI), was proposed by Chen et al. [23], was calculated according to the formula:

HPI = UFA/[C12:0 + (4 × C14:0) + C16:0]

The Desirable Fatty Acid (DFA) index was calculated according to the formula of Rhee [24] as follows:

DFA = ∑UFA + C18:0

### 2.6. Statistical Analysis

One-factor variance analysis was employed for the comparisons between the farming systems. Correlations between SCC and milk composition were calculated as Pearson’s correlations. SPSS software (version 26.0, SPSS Inc., Chicago, IL, USA) was used for data analysis. The results were considered to be significant when the *p*-values were <0.05.

## 3. Results and Discussion

### 3.1. Milk Chemical Composition

Milk from the semi-intensive system had a higher but non significantly different (*p* ≥ 0.05) ash, fat, and protein content and a lower lactose content compared to intensively produced milk (Table 1). De Renobales et al. [11] also reported that part-time grazing did not affect milk gross chemical composition in relation to milk from ewes kept indoors. However, in a similar study conducted in the same area, it was found that milk protein, fat, lactose, and ash contents were significantly lower in milk from intensive farms than in that from semi-intensive farms [25]. It is important to note that the average milk composition from both production systems has significantly lower values than the those reported by Wendorff and Haenlein [26] for milk from Greek sheep breeds (7.88% fat, 6.22% protein, 0.92% ash, and 19.43% total solids). In our study, the average lactose content was higher than the one reported in the latter study (4.42%). The average composition of milk from both production systems was within the range reported by Alichanidis et al. [27] (5.1–9% for fat, 2.5–5.1% for protein, 0.7–1% for ash, 3.7–5.5% for lactose, and 15.2–19.3% for total solids). According to data from the Hellenic Agricultural Organization [28], the average (May–June 2019) composition of milk produced in the Regional Unit of Florina where the study was conducted was as follows: 6.23% fat, 5.51% protein, 4.78% lactose, and 11.12% non-fat solids. Furthermore, countrywide, the gross composition of milk in the same period was as follows: 6.47% fat, 5.55% protein, 4.75% lactose, and 11.08% non-fat solids. There were no significant differences (*p* ≥ 0.05) in the fat/protein ratio between milk samples from the two production systems. The ratio was similar (1.199) to the one calculated considering the fat and protein content of milk from dairy ewes in Greece [4]. The fat/protein ratio has been associated with cheese quality characteristics such as texture, smoothness, and fineness in goat cheeses [29]. Finally, it is critical to bear in mind that although part-time grazing was associated with a reduction of the amount of concentrates provided to the animals, there was no adverse effect on the gross composition of the produced milk. Thus, milk production from animals on the semi-intensive system may be better from a financial point of view.

Milk somatic cell count also did not differ (*p* ≥ 0.05) in relation to the two production systems. Bulk tank somatic cell count is the first and most important index used by farmers and the dairy industry to assess udder health in the flocks and milk hygiene because increased somatic cell counts affect milk production and cheese yield. Although the European Union (Regulation 853/2004) [30] has set a legal limit at 400,000 cells/mL for cow milk, there is no limit for somatic cell count in sheep milk. According to Sevi et al. [31], sheep milk’s satisfactory hygienic and technological quality is achieved at 70,000 cells/mL (6.85 log10 cells/mL). Gonzalo [32] proposed a somatic cell count lower than 500,000 (5.70 log10 cells/mL) for good-quality milk, whereas the somatic cell count for healthy sheep mammary gland should not exceed 250,000 cells/mL (5.39 log10 cells/mL). Lianou et al. [16] also reported no differences in the somatic cell count in bulk tank milk collected from farms using the intensive or the semi-intensive production system. In the previous study, the somatic cell count expressed as geometric mean was higher in the milk from intensive farms than in that from semi-intensive farms (5.45 × 10^5^ vs. 4.50 × 10^5^ cells/mL, respectively).

### 3.2. Milk Physicochemical Properties

The physicochemical properties of milk did not differ significantly (*p* ≥ 0.05) in relation to the two production systems, as expected (Table 2). Milk physicochemical properties are affected by milk chemical composition and somatic cell count. Therefore, the lack of effect of the production system on the previously reported parameters was also reflected in the physicochemical characteristics of the milk obtained from the production systems.

Milk from both production systems had pH values within the range (6.41–6.79) reported by Pappa et al. [33]. However, electrical conductivity, refractive index, and Brix values were higher than the values reported in the review study of Park et al. [34] (refractive index 1.3492–1.3497 and electrical conductivity 3.8 mS/cm) and the study of Gelasakis et al. [35], where samples collected from individual animals were used (electrical conductivity 4 mS/cm and Brix value 4).

Milk pH is used as an indirect index for the microbiological quality of milk, and thus, titratable acidity is usually determined. The other three variables, i.e., electrical conductivity, refractive index, and Brix value, are not frequently determined. Electrical conductivity is used as a rapid method for detecting subclinical mastitis in individual animals [36]. Refractive index and Brix value are used to measure milk total solids [37]. Therefore, determining the physicochemical properties of milk could be a convenient, rapid strategy for assessing milk chemical composition [35] and monitoring the health status of the animals [38] at the farm level. Furthermore, the convenient and portable equipment required for these analyses would enable farmers to manage their flocks efficiently, and thus, collecting these data should be encouraged and promoted by both veterinarians and the dairy industry.

### 3.3. Milk Fatty Acid Composition and Nutritional Value

Sheep milk fatty acid composition is presented in Table 3. Myristic (C14:0), palmitic (C16:0), and stearic (C18:0) were the major saturated fatty acids in milk from both production systems. Oleic (C18:1 *cis-9*) and linoleic (C18:2 *n*-6 *cis*) acids were the predominant fatty acids in the monounsaturated and polyunsaturated lipid classes, respectively. The levels of linolenic (C18:2 *n*-6) and α-linolenic (C18:3 *n*-3) acids did not differ statistically (*p* ≥ 0.05) in milk form the intensive and the semi-intensive system. Grass contains elevated levels of α-linolenic (C18:3 *n*-3) acid, and this can explain the elevated levels of α-linolenic acid and its biohydrogenation intermediates such as conjugated linoleic acid (CLA) (C18:2 *cis-9 trans-11*), vaccenic acid (C18:1 *trans 11*), and stearic acid (C18:0) in the milk from ewes raised using the semi-intensive system [5]. Milk from the semi-intensive production system had a higher (*p* < 0.001) content of CLA, which possesses anti-carcinogenic, anti-atherogenic, anti-obesity, and anti-diabetic properties [39]. Milk and dairy products are the main sources of CLA acid in the human diet, and increased levels of this acid have been found in milk from pasture-grazing or grass-fed animals [40,41]. With regard to the nutritionally important long-chain PUFA, milk from ewes on the semi-intensive production system had significantly higher (*p* < 0.001) levels of arachidonic acid (C20:4 *n*-6), whereas there were no differences in the levels of eicosatrienoic (C20:3 *n*-3), eicosapentenoic (C20:5 *n*-3, EPA), and docosapentenoic (C22:5 *n*-3) acids in the milk from both production systems. Sheep milk does not contain high levels of long-chain PUFA due to the extensive biohydrogenation of these acids in the rumen [42] in general. The higher PUFA content (Table 4) observed in the milk from ewes on the semi-intensive system is attributed to grazing in mountainous areas, and it has been reported by Bravo-Lamas et al. [43]. Forage feeding can modify the fatty acid composition of ruminant milk, as reported in the study of Elgersma et al. [41]. According to Chilliard et al. [44], pasture feeding decreases the content of SFA, particularly, of fatty acids such as capric (C10:0), lauric (C12:0), and myristic acids, and it increases the content of fatty acids favorable to human health such as oleic acid, α-linolenic acid (C18:3 *n*-3), and conjugated linoleic acid (CLA) (C18:2 *cis-9 trans-11*). Furthermore, green plants are the primary dietary sources of *n*-3 PUFA in ruminants, and increased dietary levels of *n*-3 PUFA can improve fatty acid composition in ruminant milk and meat [40]. Furthermore, according to Angeles-Hernandez et al. [45], the ratio of forage to concentrates in the animal diet can significantly affect milk fatty acid profile.

The pattern of the fatty acid profile for milk from the intensive production system was similar to the one reported by De Renobales et al. [11] for dairy ewes on a concentrate-based diet. On the other hand, the pattern of fatty acid composition for milk from the semi-intensive production system was similar to the one reported by Bravo-Lamas et al. [43] for milk from ewes that grazed on valley grasslands close to the farm and were fed a small amount of concentrate. In general, ewes on the semi-intensive system produced milk with a better fatty acid profile. This is in agreement with the findings of De Renobales et al. [11] that reported improved fatty acid composition in milk from animals that were allowed to graze while receiving a concentrate supplement.

The effect of the production system on the lipid quality of milk is presented in Table 4. The content of SFA was significantly higher (*p* < 0.001) in the milk from the intensive system, whereas the contents of MUFA and PUFA were significantly higher (*p* < 0.001) in the milk from ewes on the semi-intensive system. However, there were no differences (*p* ≥ 0.05) in the medium-chain triglycerides (MCT) content in relation to the two production systems. Sheep milk is rich in MCT, which are important from a therapeutic point of view since they are easily absorbed and can be used in cases of nutrient malabsorption or undernourishment [46]. The h/H ratio was significantly higher (*p* < 0.001) in the milk from farms using the semi-intensive system. Chen and Liu [21] reported h/H ratio values of 0.50–1.29 for ewe milk. The h/H ratio is used to assess the effect of fatty acid composition on cholesterol and the risk of cardiovascular disease because it is linked to the functional activity of fatty acids in lipoprotein metabolism for the transport of plasma cholesterol. Higher values of the h/H ratio are desirable [47]. AI was significantly lower (*p* < 0.001) in milk from the semi-intensive production system than in milk from the intensive production system. In a recent review by Chen and Liu [21], it was reported that the AI for ewe milk ranges between 1.42 and 3.39. Similarly, the TI was also significantly lower (*p* < 0.001) in the milk from the semi-intensive system. The reported range for TI was 1.00–2.72 [21]. Both AI and TI were within the reported range for sheep milk proposed by Sinanoglou et al. [48] and lower than 3 for milk from both production systems. AI indicates the relationship between the sum of the major saturated fatty acids and that of the main classes of unsaturated fatty acids. In contrast, TI describes the thrombogenic potential of fatty acids, indicating their tendency to form clots in blood vessels [49]. Lower values of both indices are beneficial for human health. Pietrzak-Fiećko and Kamelska-Sadowska [50] reported higher values for AI and TI and a lower h/H ratio for sheep milk compared to milk from other mammalian species. HPI was also significantly higher (*p* < 0.001) in the milk from ewes in the semi-intensive farms. Bonano et al. [51] reported HPI values in the range of 0.16–0.28. Higher values of this index are desirable, since it is the reverse of TI [20]. Finally, the content of DFA was significantly higher (*p* < 0.001) in the milk from the semi-intensive production system. Atti et al. [52] also reported that pasture-based diets significantly improved the DFA value of sheep milk. DFA is the sum of unsaturated fatty acids and stearic acid, all considered anti-atherogenic since they reduce plasma cholesterol and triacylglycerols [21,53].

## 4. Conclusions

The data obtained in this study showed no effect of the farming system on the basic composition and physicochemical characteristics of commercially produced milk in farms using the intensive or the semi-intensive production system. However, milk from the semi-intensive production system had significantly improved fatty acid composition and lipid quality nutritional indices in relation to milk produced in the intensive farms. Differences in the composition of fatty acids and in the lipid quality indices were attributed to the inclusion of pasture in sheep diet. This nutritional information could add value to the produced dairy products and create product differentiation.

## Figures and Tables

**Table 1:** Effect of the production system on sheep milk chemical composition, fat/protein ratio, and somatic cell count.

Variable	Production System		SEM	Significance
	Intensive	Semi-Intensive		
Moisture (%)	82.71	82.41	0.273	NS
Ash (%)	0.81	0.82	0.008	NS
Fat (%)	6.40	6.63	0.230	NS
Protein (%)	5.39	5.53	0.097	NS
Lactose (%)	4.70	4.62	0.062	NS
Total solids	17.30	17.59	0.273	NS
Fat: Protein ratio	1.189	1.199	0.039	NS
SCC (log_10_ cells/mL)	3.271	3.288	0.100	NS

NS = Non-significant.

**Table 2:** Effect of the production system on sheep milk physical properties.

Variable	Production System		SEM	Significance
	Intensive	Semi-Intensive		
pH	6.70	6.70	0.027	NS
Electrical Conductivity (mS/cm)	4.42	4.47	0.065	NS
Refractive index	1.354	1.354	0.000	NS
Brix (°Bx)	13.69	13.73	0.161	NS

NS = Non-significant.

**Table 3:** Effect of the production system on sheep milk fatty acid composition (% of total identified fatty acids).

Fatty Acid	Production System		SEM	Significance
	Intensive	Semi-Intensive		
Saturated fatty acids				
C4:0	1.60	1.58	0.045	NS
C6:0	1.84	1.78	0.063	NS
C8:0	6.89	6.27	0.282	***
C10:0	4.65	4.06	0.213	***
C12:0	2.58	2.57	0.124	NS
C14:0	12.50	11.44	0.304	***
C15:0	1.12	1.08	0.040	NS
C16:0	29.55	26.57	0.628	***
C17:0	0.70	0.80	0.033	***
C18:0	8.73	11.09	0.579	***
C20:0	0.32	0.36	0.040	NS
C21:0	0.18	0.17	0.014	NS
C22:0	0.03	0.04	0.008	NS
Monounsaturated fatty acids				
C14:1	0.46	0.49	0.019	NS
C15:1	0.29	0.29	0.014	NS
C16:1	1.20	1.02	0.056	***
C17:1	0.29	0.29	0.016	NS
C18:1 *trans*	0.50	0.54	0.052	NS
C18:1 *trans-11 (VA)*	1.59	1.78	0.221	NS
C18:1 *cis-9*	20.19	22.47	0.669	***
C20:1	0.11	0.15	0.024	NS
Polyunsaturated fatty acids				
C18:2 *n*-6 *cis*	2.66	2.54	0.112	NS
C18:2 *n*-6 *trans*	0.82	1.13	0.084	***
C18:3 *n*-3	0.66	0.78	0.072	NS
C18:2 *cis-9 trans-11* (CLA)	0.30	0.42	0.054	***
C20:2	0.05	0.05	0.007	NS
C20:4 *n*-6	0.10	0.12	0.009	***
C20:3 *n*-3	0.03	0.04	0.008	NS
C20:5 *n*-3	0.06	0.08	0.010	NS
C22:5 *n*-3	0.01	0.01	0.004	NS

NS = Non-significant; *** = *p* < 0.001.

**Table 4:** Effect of the production system on sheep milk nutritional indices.

Index	Production System		SEM	Significance
	Intensive	Semi-Intensive		
SFA ^1^	70.69	67.81	0.772	***
MUFA ^2^	24.64	27.02	0.728	***
PUFA ^3^	4.68	5.17	0.237	***
Medium chain triglycerides (C6:0–C14:0)	27.86	26.94	0.919	NS
h/H ^4^	0.560	0.688	0.027	***
AI ^5^	2.825	2.355	0.115	***
TI ^6^	2.915	2.513	0.100	***
HPI ^7^	0.359	0.435	0.018	***
DFA ^8^	38.038	43.279	1.215	***

^1^ = Saturated fatty acids; ^2^ = Monounsaturated fatty acids; ^3^ = Polyunsaturated fatty acids; ^4^ = Hypocholesterolemic/Hypercholesterolemic index; ^5^ = Atherogenicity index; ^6^ = Thrombogenicity index; ^7^ = Heath promoting index; ^8^ = Desirable fatty acids; *** *p* < 0.001; NS = Non-significant.

## Data Availability

The data presented in this study are contained within the article.

## Abbreviations

AI	Atherogenicity index
CLA	Conjugated fatty acids
DFA	Desirable fatty acids
EFSA	European Food Safety Authority
h/H	Hypocholesterolemic/hypercholesterolemic ratio
HPI	Health-promoting index
MCT	Medium chain triglycerides
MUFA	Monounsaturated fatty acids
PUFA	Polyunsaturated fatty acids
SFA	Saturated fatty acids
TI	Thrombogenicity index
VA	Vaccenic acid
